# Follicle-stimulating hormone promotes the proliferation of epithelial ovarian cancer cells by activating sphingosine kinase

**DOI:** 10.1038/s41598-020-70896-0

**Published:** 2020-08-14

**Authors:** Keqi Song, Lan Dai, Xiaoran Long, Wenjing Wang, Wen Di

**Affiliations:** 1 Department of Obstetrics and Gynecology, Ren Ji Hospital, School of Medicine, Shanghai Jiao Tong University, Shanghai, 200127 China; 2 Shanghai Key Laboratory of Gynecologic Oncology, Ren Ji Hospital, School of Medicine, Shanghai Jiao Tong University, Shanghai, 200127 China; 3 State Key Laboratory of Oncogene and Related Genes, Shanghai Cancer Institute, Ren Ji Hospital, School of Medicine, Shanghai Jiao Tong University, Shanghai, 200127 China

**Keywords:** Cancer, Oncology

## Abstract

Follicle-stimulating hormone (FSH) is closely related to the pathogenesis and progression of epithelial ovarian cancer (EOC). However, until now, knowledge relating to FSH-driven signalling pathways that lead to the growth of EOC remained incomplete. We sought to explore whether sphingosine kinase (SphK) could mediate FSH-induced ovarian cancer cell proliferation and which pathway might be involved in this process. The expression of phospho-SphK1 and phospho-SphK2 was detected in sections of EOC tissues by Immunohistochemical staining, and clinical significances were analyzed by statistical analysis. EOC cells were treated with FSH or/and SKI-II. CCK8 assays and colony formation assays were used to investigate cell proliferation. Western blot was carried out to detect protein expression in EOC cell line after treated with FSH. Here, for the first time, we provide evidence that high expression levels of phospho-SphK1 and phospho-SphK2 were both prognostic indicators of overall survival (OS) in EOC. Additionally, the expression levels of both phospho-SphK1 and phospho-SphK2 were closely correlated with the expression level of follicle-stimulating hormone receptor (FSHR) in ovarian cancer tissues. FSH stimulated the phosphorylation of both SphK1 and SphK2 and was able to regulate the survival and growth of ovarian cancer cells by activating SphK1 and SphK2 through ERK1/2. Both isoenzymes of SphK were equally responsible for FSH-induced cell proliferation of EOC. Both Erk1/2 and Akt activation play important roles in mediating FSH-induced cell proliferation after phosphorylation of SphK. Moreover, our data demonstrated that S1P receptor 1 (S1PR1) and S1PR3, key components of the SphK signalling system, were involved in FSH-mediated proliferation of EOC. Taken together, the results of the current study revealed that SphK is an essential mediator in FSH-induced proliferation of ovarian cancer cells in EOC, which indicates a new signalling pathway that controls FSH-mediated growth in EOC and suggests a new strategy that pharmaceutically targets both isoenzymes of SphK for the management of ovarian cancer.

## Introduction

Among all gynaecological malignancies, ovarian cancer is the leading cause of death. In the past few decades, the incidence of ovarian cancer has continued to increase. Ovarian cancer accounts for approximately 23% of all tumours of the female reproductive system tumor, but its mortality rate is 47%. In 2019, it is expected that, in the United States, 22 530 women will suffer from ovarian cancer and 13 980 will die due to ovarian cancer in United States^1^.

The molecular mechanism relating to the origin and progression of ovarian cancer remains unclear. Previous studies showed that 80–90% of ovarian cancers originate from the ovarian surface epithelium, and multiple reports have confirmed the involvement of endocrine factors in the tumorigenesis of epithelial ovarian cancer (EOC). In both epidemiological and clinical studies, increasing evidence has indicated a strong association between high levels of FSH and an increased incidence of EOC^2–5^. In addition, the average age of onset for EOC is 50–60 years. The level of FSH in women at this age is particularly high, reaching almost 10–20 times that in women of reproductive age^4^. Epidemiologic studies show that there is a clear trend of significantly decreasing risk of EOC with increasing numbers of pregnancies and deliveries, extended duration of oral contraceptive use, and extended duration of breastfeeding, all of which are associated with lower levels of and reduced exposure to FSH^6^. Studies focusing on the role of FSH have found that, in vitro, it could not only stimulate the growth of normal or immortalized ovarian surface epithelial cells but also accelerate some ovarian cancer cell growth in a time-dependent and dose-dependent manner. Additionally, different signalling pathways have been implicated in FSH-induced malignant biological activities in EOC cells, such as activation of mitogen-activated protein kinases (MAPKs), protein kinase A (PKA), phosphoinositide 3-kinase (PI3K), and protein kinase C (PKC)^7^. External stimuli, such as hormones (i.e., FSH)^8^ and stress (i.e., muscle injury)^9^ could activate through those pathways in cells and further promote cells proliferation and differentiation. Meanwhile, chemotherapeutic drugs (i.e., cisplatin) could alter those pathways to induce cell apoptosis^10^. In the study of Choi, it was reported that treatment with FSH resulted in activation of the MAPK cascade and activated MAPK-phosphorylated Elk-1 in immortalized ovarian surface epithelium (IOSE) cell lines (normal), IOSE-29 (preneoplastic) and IOSE-29EC (neoplastic and tumorigenic)^11^. These results indicated that FSH exerted a growth stimulatory effect in normal, preneoplastic, and neoplastic OSE cells via MAPK pathway^11^. However, other mechanisms responsible for the growth effect of FSH on EOC remains to be identified. A deeper understanding of the molecular basis of the effects of FSH in ovarian cancer is required to establish innovations in the therapeutic approach.

Sphingosine kinase (SphK) is considered an important signalling enzyme in the sphingolipid metabolic pathway and can catalyse the phosphorylation of sphingosine to produce sphingosine-1-phosphate (S1P). SphK is expressed as two isoforms, SphK1 and SphK2, with SphK1 emerging as an important and critical signalling enzyme for promoting cell growth, inhibiting apoptosis and facilitating oncogenesis^12^. Consistent with our previous study, it was found that in many human cancers, including EOC, SphK1 expression was significantly increased and promoted cancer development via cancer angiogenesis and metastasis^13,14^. Rosales-Torres showed that FSH increased S1P synthesis in granulosa cells by phosphorylating SphK1^15^. Regarding SphK2, its role is relatively complex because both pro-apoptotic and pro-proliferative functions have been suggested. Despite this, SphK2 has been recognized as overexpressed in many human cancers, and low-level SphK2 overexpression has been shown to promote cell proliferation and survival^16^. An inhibitor of SphK2, ABC294640, displays anti-EOC activities both in vitro and in vivo^17^. This indicates that SphK2 also promotes ovarian cancer. Earlier studies showed that the key to affecting the enzyme activity of SphK was the phosphorylation of SphK1 and SphK2, and this process relied on activation of the Erk pathway^18,19^. At present, it is still unknown whether these two isoforms of SphK have a synergistic or antagonistic function in human cancer. In addition, whether FSH-induced activation of the Erk pathway in ovarian cancer cells causes variation in SphK1 and SphK2 expression and activity is still unclear.

SphK catalyses the formation of S1P, which regulates cell behaviour by interacting with sphingosine-1-phosphate receptor (S1PR)^20^. We confirmed in a previous study that the expression levels of S1PR1 and S1PR3 were significantly increased in EOC^13^. The SphK/S1PR signalling pathway was considered to interact with a complex growth factor network and facilitate cancer cell proliferation. Nonetheless, the network relating to SphK/S1PR regulation of EOC proliferation and whether this network mediates FSH-induced proliferation in EOC has not been well defined.

The purpose of our study was to evaluate the relationship between the expression of SphK and FSH receptor (FSHR) in EOC, to evaluate the role of SphK1 and SphK2 in FSH-driven progression of EOC cells, and to explore the underlying mechanisms in EOC cell survival.

## Material and methods

### Tissue samples

Tissue samples were collected from patients with primary EOC, including 24 patients with stage I-II disease and 34 with stage III-IV disease, who were undergoing surgical treatment without preoperative chemotherapy. 57 patients were followed up, 1 case lacked follow-up data. This study was approved by the Institutional Review Board of Shanghai Jiaotong University. All patients provided informed consent.

### Immunohistochemical staining

The specific experimental methods used for immunostaining were performed as previously described^17^. Three slides of each sample were prepared and examined. Polyclonal antibodies against phospho-SphK1 (Ser225) (anti-rabbit, 1:100, ECM bioscience KY), phospho-SphK2 (Thr-578) (anti-rabbit, 1:100, ECM bioscience KY), and FSHR (anti-rabbit, 1:100, Abcam USA) were used as primary antibodies. Stained sections were observed under a microscope. Immunostaining was observed by two independent experienced pathologists who were blinded to the clinicopathological parameters and clinical outcomes of the patients. Evaluation of the intensity of immunostaining, the proportion of positively stained tumour cells and the staining index (SI) were performed as described in our previous study^13^. A final score ≥ 4 was used to indicate tumours with high expression of phospho-SphK1, phospho-SphK2 or FSHR, and a score ≤ 3 reflected low expression. The optimal cut-off value for high and low expression of phospho-SphK1 or phospho-SphK2 was chosen on the basis of the distribution of the staining results and a measure of heterogeneity with the log-rank test with respect to overall survival (OS).

### Cell lines and cell culture

The human EOC cell line HO8910 was obtained from the Cell Bank of Chinese Academy of Sciences (Shanghai, China) and routinely cultured in RPMI 1,640 (HyClone, USA) supplemented with 10% foetal bovine serum (Invitrogen) and 1% antibiotics. HEY cells were purchased from American Type Culture Collection (VA, USA) and routinely cultured in DMEM (HyClone, USA). The cells were tested to confirm no mycoplasma or cross-contamination. All cells used in experiments underwent no more than 20 passages.

### Reagents and antibodies

The SphK inhibitor (SKI-II), MEK1/2 inhibitor (U0126) and SphK2 inhibitor (ABC294640) were purchased from Selleck Company (Shanghai, China) and dissolved in DMSO for storage until further use. The SphK1 inhibitor (PF543) was purchased from MCE Company (Shanghai, China) and dissolved in DMSO for storage until further use. The S1PR1/3 inhibitor (VPC29013) was purchased from SantaCruz Company (Dallas, USA) and dissolved in DMSO for storage until further use. Antibodies against GAPDH, FSHR, SphK1 and SphK2 were purchased from Abcam (Cambridge, UK). Antibodies against phospho-Erk1 (Thr202/Tyr204)/phospho-Erk2 (Thr185/Tyr187), Erk1/2, phospho-Akt (Ser473) and Akt were purchased from Cell Signaling Technology (Shanghai, China).

### Cell viability assay

The viability of cells after different treatments was measured by the CCK-8 assay from Dojindo (Kumamoto, Japan) as described in our previous study^17^. HEY and HO8910 cells were seeded in a 96-well plate at a density of 3 × 10^3^ cells in each well. After the cells were serum starved for 24 h, they were treated with FSH (SKI-II or FSH/SKI-II) for 72 h. The optical densities (OD) were read at 450 nm.

### Colony formation assay

The specific experimental methods were performed as previously described^21^. After serum starvation for 24 h, cells were treated with FSH in the presence or absence of SKI-II in DMEM supplemented with 1% foetal bovine serum. Clonogenic cell survival was normalized to that of DMSO vehicle control.

### Small interfering RNA (siRNA) and transient transfection

siRNAs targeting human SphK1 (5′AAGAGCUGCAAGGCCUUGCCC-3′), SphK2 (5ʹ-AACCUCAUCCAGACAGAACGA-3ʹ), FSHR (5′AGAAAAACCCUGUUAGAGCAG3′), S1PR1 (5′-AAGCUACACAAAAAGCCUGGA-3′), S1PR2 (5′-AAUACCUUGCUCUCUGGCUCU-3′), and S1PR3 (5′-CUGCCUGCACAAUCUCCCUTT-3′) in addition to a scrambled control siRNA (5 ‘-AAUUCUCCGAACGUGUCACGU-3′) were synthesized by GenePharma (Shanghai, China). siRNA duplexes were transfected using Lipofectamine 2000 (Invitrogen, USA) according to the manufacturer’s protocol. After 24 h of transfection, the levels of the targeted genes were detected by qRT-PCR. Forty-eight hours later, the levels of the targeted genes were detected by Western blots.

### Quantitative real-time PCR

TRIzol reagent (Invitrogen, USA) was used to isolate total RNA. The mRNA levels were measured by SYBR Green RT-PCR and calculated by the 2-ΔΔCt method. The primers for specific genes were as follows: FSHR, 5′-GCATTCAATGGAACCCAACTAG-3′ (forward) and 5′-CGTGGAAAACATCATTAGGCAAS1PR1-3′ (reverse); SphK1, 5′-CATTATGCTGGCTATGAGCAG-3′ (forward) and 5′-GTCCACATCAGCAATGAAGC-3′ (reverse); SphK2, 5′-GGTTGCTTCTATTGGTCAATCC-3′ (forward) and 5′-GTTCTGTCGTTCTGTCTGGATG-3′ (reverse); S1PR1, 5′-CCTCTTCCTGCTAATCAGCG-3′ (forward) and 5′-ACAGGTCTTCACCTTGCAGC-3′ (reverse); S1PR2, 5′-CATTGCCAAGGTCAAGCTGT-3′ (forward) and 5′-ACGATGGTGACCGTCTTGAG-3′ (reverse); S1PR3, 5′-TCAGCCTGTCTCCCACGGTC-3′ (forward) and 5′-ACGGCTGCTGGACTTCACCA-3′ (reverse); and GAPDH, 5′-TGCACCACCAACTGCTTAGC-3′ (forward) and 5′-GGCATGGACTGTGGTCATGAG-3′ (reverse).

### Western blot analysis

Cells were harvested after the indicated treatments. The protein concentration was determined using BCA reagent. Western blotting was performed as previously described^21^.

### Statistical analysis

The SPSS 24.0 software package (SPSS, Inc., Chicago, IL) was used to perform the statistical analyses in our study. The statistical significance of the correlations between phospho-SphK1/phospho-SphK2/FSHR expression and the clinicopathological features were analysed using the Chi-square test. The relationship between phospho-SphK1 or phospho-SphK2 and FSHR expression was examined using the Pearson correlation test. For analyses of the survival data, Kaplan–Meier curves were constructed, and log-rank tests were performed. The values are presented as the means ± SDs, and a t-test was used for the statistical analysis (*P*  < 0.05 was considered significant).

### Ethics approval and consent to participate

This study was conducted in accordance with the Declaration of Helsinki principles. It was approved by the Medical Research Ethics Committee of Renji Hospital, School of Medicine, Shanghai Jiao Tong University.

### Consent for publication

Manuscript is approved by all authors for publication.

## Results

### Elevated levels of phospho-SphK1 and phospho-SphK2 are accompanied by increased FSHR expression in ovarian cancer tissues

**Figure 1:**
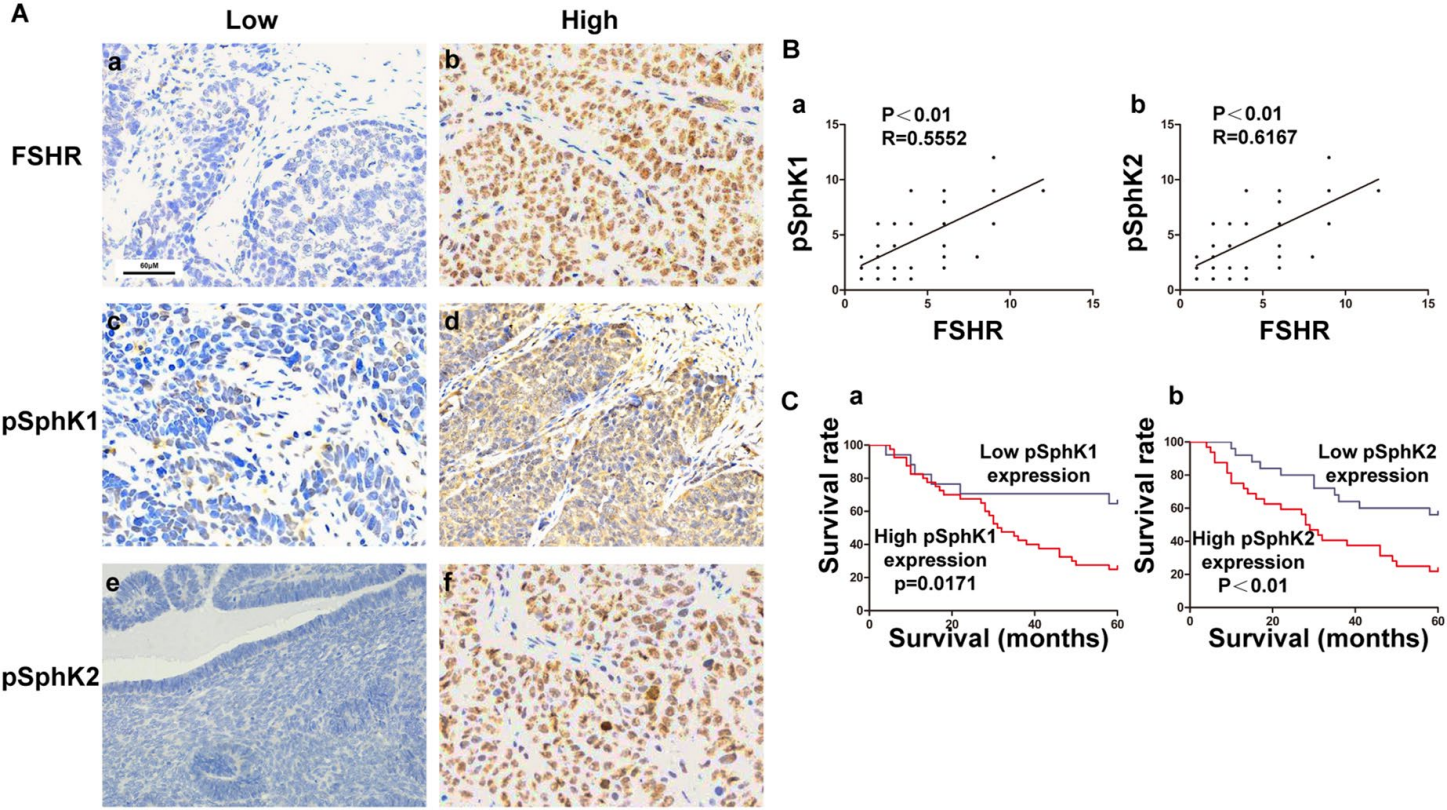
EOC tissues showed elevated expression of phosphor-SphK1 and phosphor-SphK2. (**A**) Immunohistochemical staining for pSphk1, pSphk2 and FSHR in EOC tissues. (a) Low FSHR expression; (b) high FSHR expression; (c) low phosphor-Sphk1 expression; (d) high phosphor-Sphk1 expression high phosphor-Sphk2 expression; (e) low phosphor-Sphk2 expression; (f) high phosphor-Sphk2 expression. Magnification,200 × (a–f). (**B**) The relationship between expression of pSphk1 (pSphk2) and FSHR in epithelial ovarian cancer tissues. We used Pearson’s correlation coefficient to detect the correlation between the variables of pSphk1and FSHR (a) (r = 0.5552, *P*  < 0.01), pSphk2 and FSHR (b) (r = 0.6167, *P*  <01) as indicated by the regression line. (**C**) Kaplan–Meier survival analysis of the overall survival of epithelial ovarian cancer patients. (a) High phosphor-Sphk1 expression. (b) High phosphor-Sphk2 expression. *P* < 0.05 based on the log-rank test.

We examined phospho-SphK1, phospho-SphK2, and FSHR expression in 58 samples from EOC patients. Figure 1Aa,Ac,Ae show representative photographs of tissues with low levels of FSHR, phospho-SphK1, and phospho-SphK2 expression. Figure 1Ab,Ad,Af show representative photographs of elevated levels of FSHR, phosphor-SphK1 and phosphor-SphK2, respectively. We examined the relationship between phospho-SphK1/phospho-SphK2 expression levels in EOC and clinicopathological characteristics (Table 1). High levels of phospho-SphK1/ phospho-SphK2 were associated with higher FIGO stage**.** In the current study, we found that both phospho-SphK1 and phospho-SphK2 were significantly correlated with the FSHR level in tumour tissue (Fig. 1B).

**Table 1:** Clinicopathological features of ovarian tissue with regard to the relative expression of pSphK1 and pSphK2.

Parameters	No. Case	pSphK1 expression				pSphK2 expression			
		Low	High	*P* ^a^	χ^2^	Low	High	*P* ^a^	χ^2^
**Age (years)**									
≤ Mean	23	7	16	0.694	0.155	10	13	0.963	0.002
> Mean	35	11	24			15	20		
**FIGO disease stage**									
I-II	23	10	13	0.028*	4.819	14	9	0.027*	4.905
III-IV	35	6	29			11	24		
**Histologic grade**									
I	9	2	7	0.695	0.153	5	4	0.412	0.674
II–III	49	14	35			20	29		
**Lymph node metastasis**									
Absent	22	5	17	0.517	0.419	9	13	0.792	0.070
Present	36	11	25			16	20		
**Ascites**									
Absent	23	6	17	0.836	0.043	9	14	0.620	0.245
Present	35	10	25			16	19		

*P* values are calculated by χ^2^ test or Fisher’s exact test.

### High phospho-SphK1 and phospho-SphK2 levels correspond to a lower postoperative 5-year OS

Adequate clinical follow-up information was available for all 57 patients with ovarian cancer. The prognostic value of phospho-SphK1 and phospho-SphK2 was analysed by comparing the OS of patients with high and low SphK2 expression. For both phospho-SphK1 and phospho-SphK2, Kaplan–Meier analysis showed that patients with high expression had a significantly lower postoperative 5-year OS than patients with low expression (Fig. 1Ca, *P* < 0.05 and Fig. 1Cb, *P* < 0.01).

Thus, we predicted that FSHR overexpression would lead to increases in phospho-SphK1 and phospho-SphK2 levels, which would contribute to the progression of EOC.

### The proliferation of FSH-stimulated EOC cells is dependent on SphK activity

**Figure 2:**
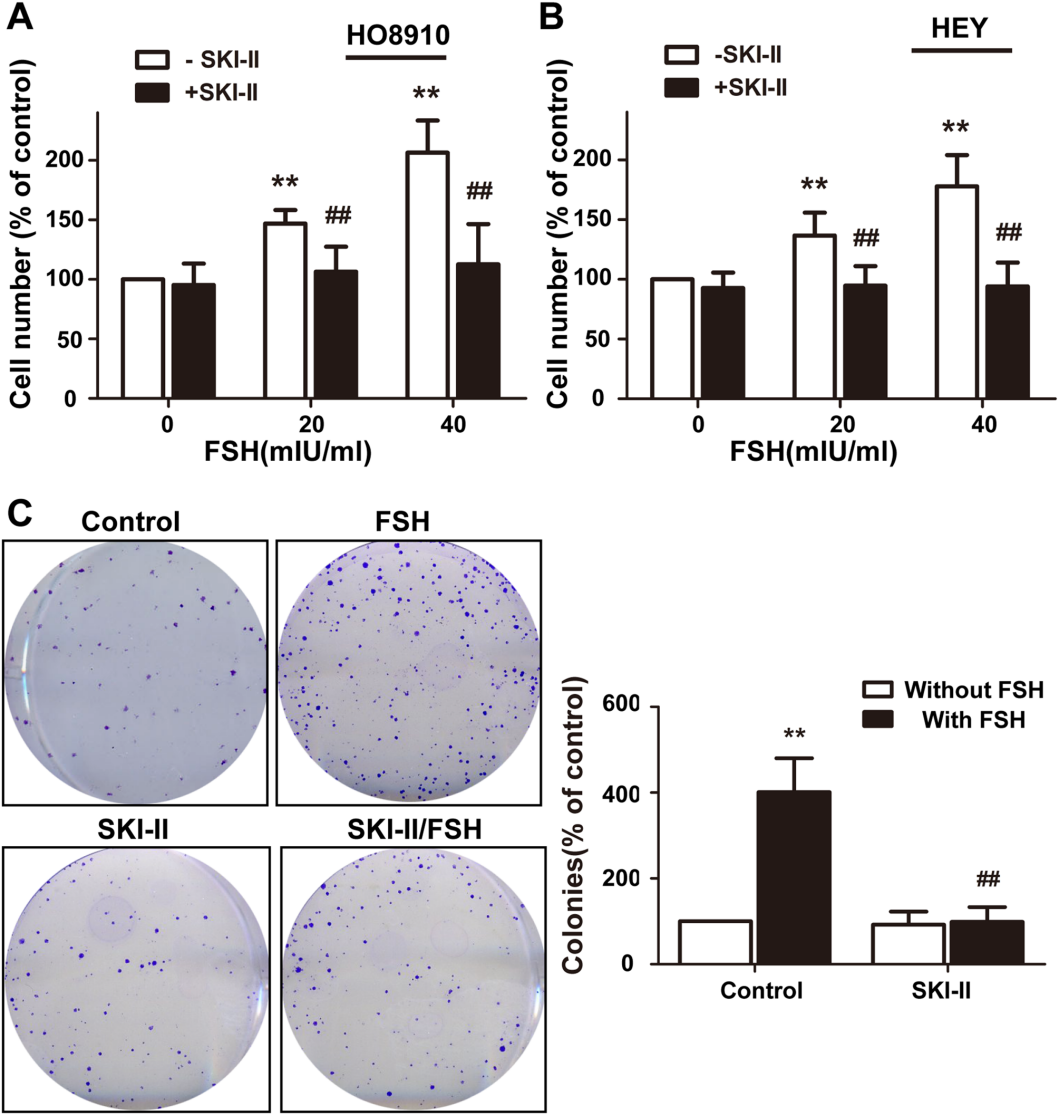
Inhibition of SphK abolished EOC cells proliferation stimulated by FSH. (**A**) HO8910 cells were serum-starved for 24 h and stimulated by FSH (40mIU/ml) with or without SKI-II (2.5 µM) for 72 h. Cell growth was measured using the CCK-8. (**B**) The same treatment was performed in cell line HEY. (**C**) HO8910 was plated in six-well plates reached 1,000 cells/well and serum-starved. Then, cells were cultured with FSH (40mIU/ml) with or without SKI-II (1 µM) in medium supplemented with 1% fetal bovine serum. Colonies were counted at the end of 2 weeks. Data are mean ± SD. *P < 0.05, vs. control; #*P* < 0.05, vs. FSH alone.

To determine whether FSH-mediated proliferation of EOC cells occurs through a pathway that involves SphK activation, SKI-II, a highly SphK-selective inhibitor, was used to block SphK activity in EOC cells^22^. We then measured the proliferation of EOC cells treated with FSH. Consistent with previous reports^23,24^, treatment with FSH at doses of 20 mIU/ml and 40 mIU/ml for 72 h led to a significant increase in HO8910 cell proliferation (Fig. 2A). In another human ovarian cancer cell line, HEY, similar results were obtained (Fig. 2B). At the same time, we realized that SKI-II almost completely abolished FSH-induced proliferation of EOC cells. As the control, treatment with SKI-II alone did not significantly inhibit cell growth (Fig. 2A,B). To further determine the effect of SphK on FSH-mediated cell proliferation of EOC, we assessed the colony-forming abilities of the treated cells to evaluate the long-term effect. As shown in Fig. 2C, FSH treatment significantly promoted the colony-forming ability of HO8910 cells, whereas treatment with both FSH and SKI-II profoundly suppressed the accelerated proliferation driven by FSH.

Based on the observed short-term and long-term survival activity, it was thought that SphK was critically involved in the FSH-stimulated proliferation of EOC cells.

### Both SphK1 and SphK2 are activated by FSH stimulation via Erk1/2 in EOC cells

**Figure 3:**
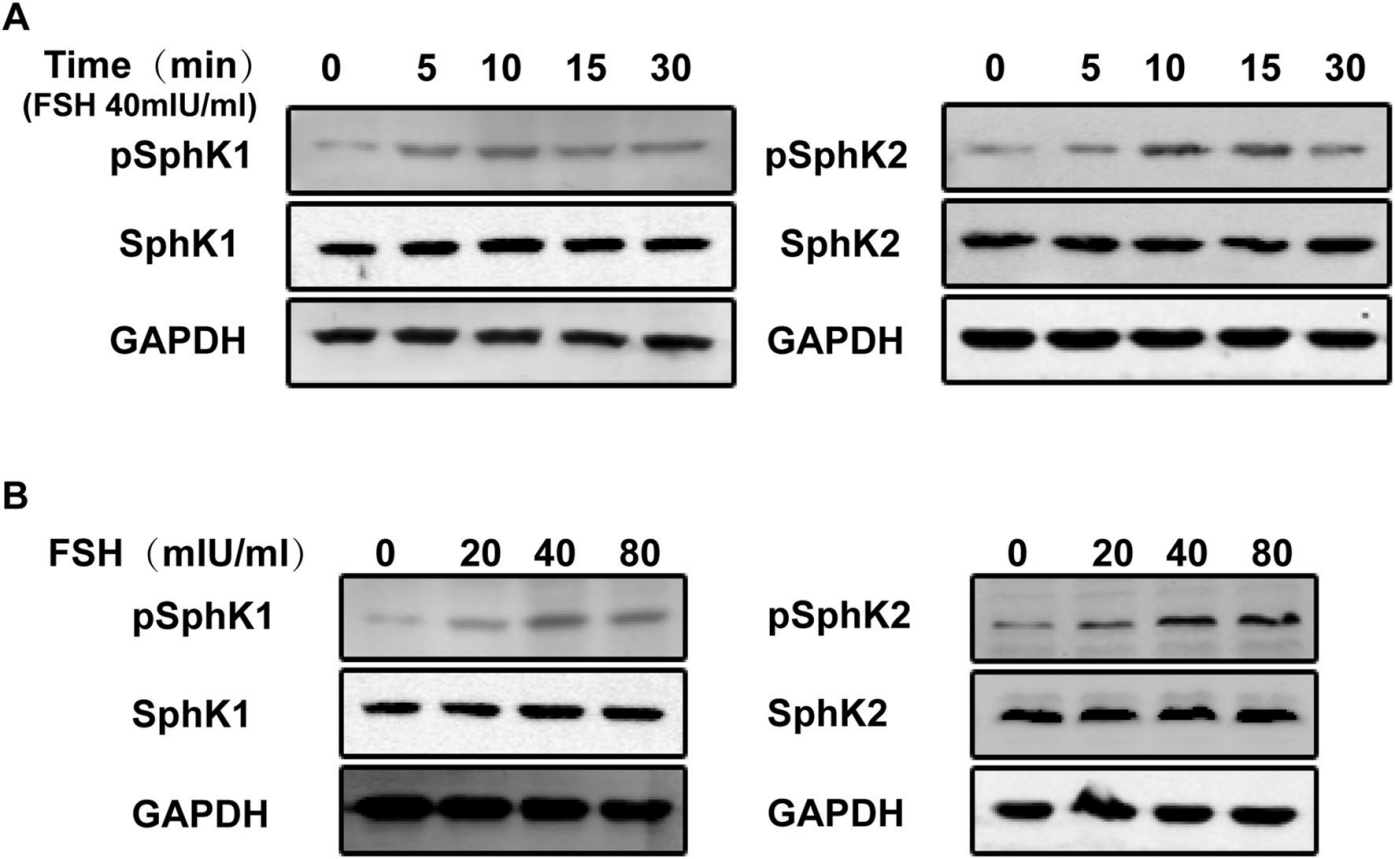
Stimulation of FSH activated phosphorylation of SphK, and raised its activity of SphK in EOC cells. (**A**) FSH stimulated serum starved HO8910 cells for the indicated time. Immunoblotting analysis with specific anti-phosphorylated SphK1 (pSphK1) and pSphK2 antibodies was performed to detect the activity of SphK1 and SphK2. The histogram showed the densitometric analysis of pSphK1 and pSphK2 (normalized to SphK1 and SphK2). (**B**) Serum-starved HO8910 cells were treated with FSH at indicated doses. After 15 min stimulation, pSphK1 and pSphK2 were determined by immunoblotting analysis. Data are mean ± SD. *P < 0.05, vs. control.

Given the potential role of SphK in FSH-stimulated proliferation, we explored whether FSH could activate SphK. According to previous reports, it is clear that phosphorylation at Ser225 of SphK1 and at Thr578 of SphK2 is key to activating the respective enzymes^18,19^. Because both SphK1 and SphK2 affected the activity of SphK in cells, we observed the phosphorylation status of SphK1 and SphK2 and examined the effect of FSH on both SphK isoforms. As shown in Fig. 3, in HO8910 cells, FSH stimulation induced a transient and rapid increase in phosphorylation at Ser225 of SphK1 and at Thr578 of SphK2. The increase in phosphorylation induced by FSH was time-dependent, as shown in Fig. 3A, with phosphorylation of SphK1 peaking within 10 min of FSH treatment and phosphorylation of SphK2 peaking within 15 min. FSH-induced phosphorylation of two isoforms of SphK showed a similar temporal response, peaked at almost 10 min and then declined. Moreover, FSH-induced phosphorylation of both isoforms of SphK in HO8910 cells showed similar dose-dependent trends, with the maximum response observed at 40 mIU/ml FSH (Fig. 3B).

**Figure 4:**
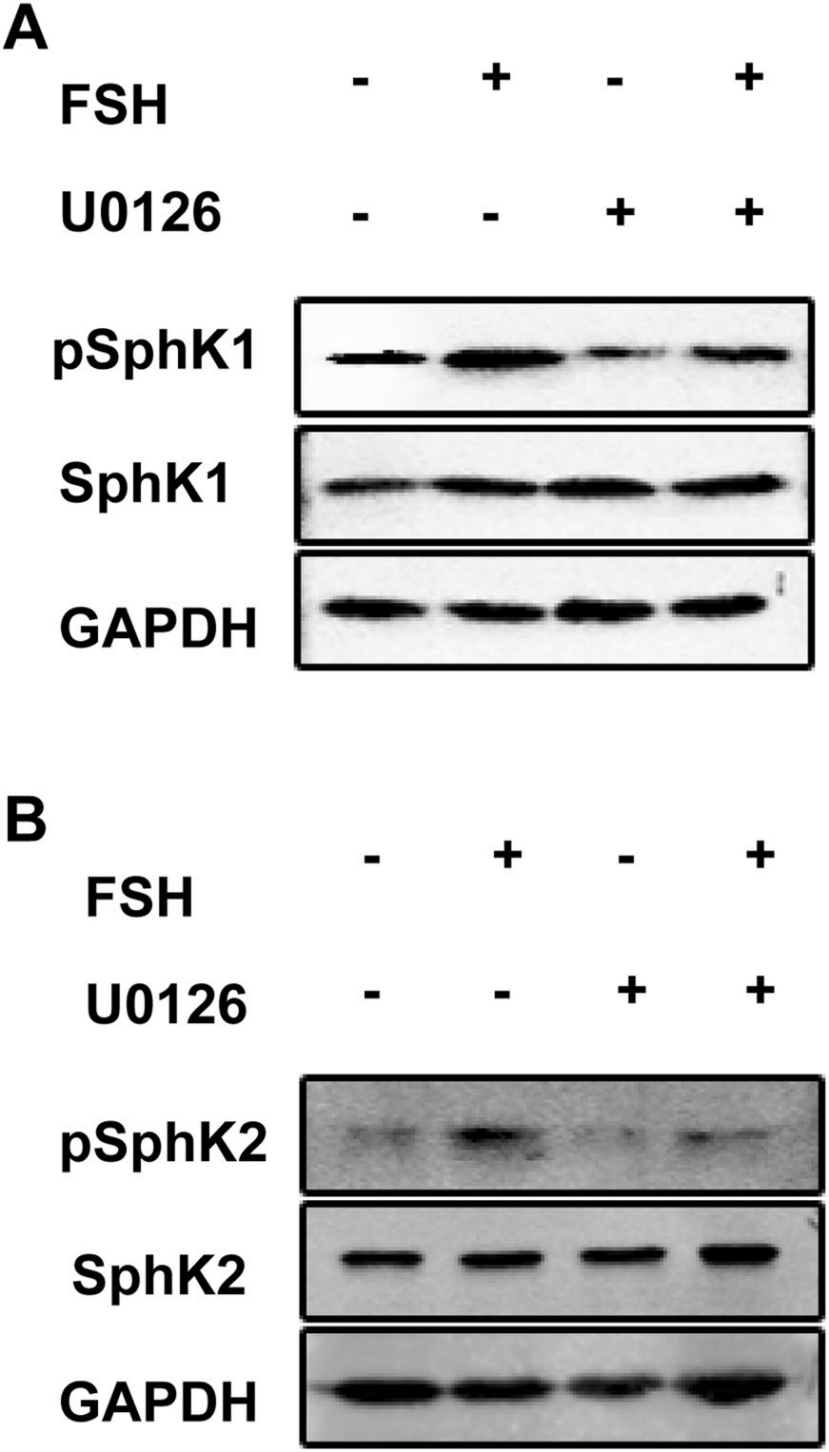
FSH stimulated increase of phosphorylation for SphK1 and SphK2 via Erk dependent pathway. Serum-starved HO8910 cells were pretreated by U0126 (5 µM) for 2 h. And then cells were treated with or without 40mIU/ml FSH. (**A**) At the end of 15 min, levels of pSphK1 were determined by Immunoblotting analysis. Densitometric analysis corresponding to the bands for pSphK1 was shown in the bottom panel (normalized to SphK1 and SphK2, respectively). (**B**) Levels of pSphK2 were determined by immunoblotting analysis. Data are mean ± SD. *P < 0.05.

Previous studies indicated that activation of the Erk pathway is considered a key factor that increases SphK1 and SphK2 phosphorylation^18,19^. In our study, we confirmed this finding and also found that the FSH-induced increase in SphK1 and SphK2 phosphorylation in HO8910 cells was completely blocked by U0126, a specific inhibitor of the Erk1/2 pathway (Fig. 4A,B). Similar results were also observed in HEY cell line (data not shown).

Taken together, these data indicated that FSH activates both SphK1 and SphK2 in an Erk-dependent manner in EOC cells.

### Both SphK1 and SphK2 are responsible for FSH-induced proliferation in EOC cells

**Figure 5:**
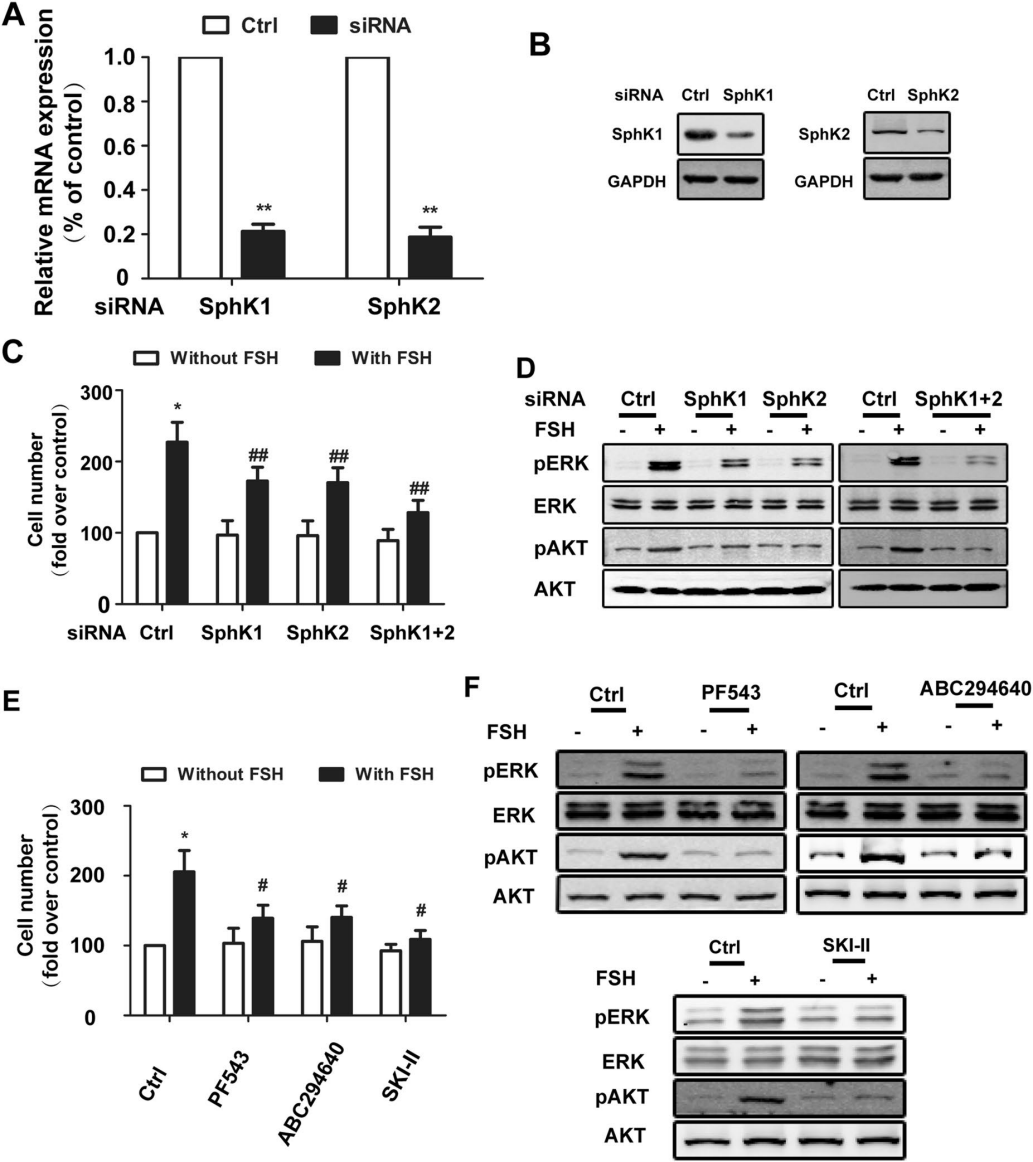
SphK1 and SphK2 played equal roles on cell proliferation stimulated by FSH. (**A**) HO8910 cells were transfected with control siRNA or siRNA targeting SphK1 or SphK2. After 24 h of transfection, mRNA levels of SphK1 and SphK2 were determined by quantitative RT-PCR. Data are expressed as the change with respect to control siRNA. (**B**) After 48 h of transfection, protein levels of SphK1 and SphK2 were determined by immunoblotting analysis and normalized to GAPDH. The data were expressed as changes relative to the control siRNA. (**C**) Having been transfected with the different siRNA, HO8910 cells were cultured with or without FSH (40 mIU/ml). After 72 h, cell survival ability was detected. (**D**) HO8910 cells transfected with specific siRNA were treated with FSH (40 mIU/ml) for 30 min. Phosphorylated-Erk (pErk) and phosphorylated Akt (pAkt) were observed by Immunoblotting analysis. (**E**) Serum-starved HO8910 cells were pretreated by different SphK inhibitors (SKI-II 2.5 µM, PF543 25 µM, ABC294640 50 µM) for 2 h. And then HO8910 cells were cultured with or without FSH (40 mIU/ml). After 72 h, cell survival ability was detected. (**F**) HO8910 cells, having been pretreated with different SphK inhibitors, were treated with FSH (40 mIU/ml) for 30 min. Phosphorylated-Erk (pErk) and phosphorylated Akt (pAkt) were observed by immunoblotting analysis. Data are mean ± SD. *P < 0.05, vs. control/-FSH; #*P* < 0.05, vs. control/ + FSH.

Having confirmed that FSH could promote the proliferation of EOC cells by activating SphK, we attempted to further study the role of each SphK isoform on FSH-mediated proliferation of EOC cells. Specific siRNAs targeting SphK1 or SphK2 were transfected into EOC cells, which resulted in an efficient reduction in the respective mRNA and protein levels compared to those in control cells transfected with a negative control vector (Fig. 5A,B). As shown in Fig. 5C, in cells with SphK1 and SphK2 knockdown, FSH-mediated proliferation was both significantly inhibited and reduced (Fig. 5C). After inhibition of SphK activity with inhibitors, we also obtained a similar trend (Fig. 5E). These results indicated that SphK1 and SphK2 are involved in FSH-mediated proliferation.

To investigate the underlying molecular mechanisms whereby SphK is required for the growth of EOC cells, we performed Western blot analysis. The Erk and Akt pathways are two established downstream signalling pathways involved in FSH-induced proliferation^24–26^. We noticed that the Erk and Akt pathways in HO8910 cells were significantly activated after increased SphK activity. However, in cells with SphK1 and SphK2 blockage, the increases in the phosphorylation of Erk and Akt were dramatically reduced (Fig. 5D,F). Remarkably, knocking down both SphK1 and SphK2 expression resulted in the most significant inhibition of ERK and AKT phosphorylation in cells stimulated by FSH (Fig. 5D). Considering the results of proliferation assays in Fig. 5C, we speculated that SphK1 and SphK2 have an additive effect on the proliferation of HO8910 cells. Similar results were also observed in HEY cell line (data not shown).

### S1PR1 and S1PR3 are responsible for the proliferation stimulated by FSH in EOC

**Figure 6:**
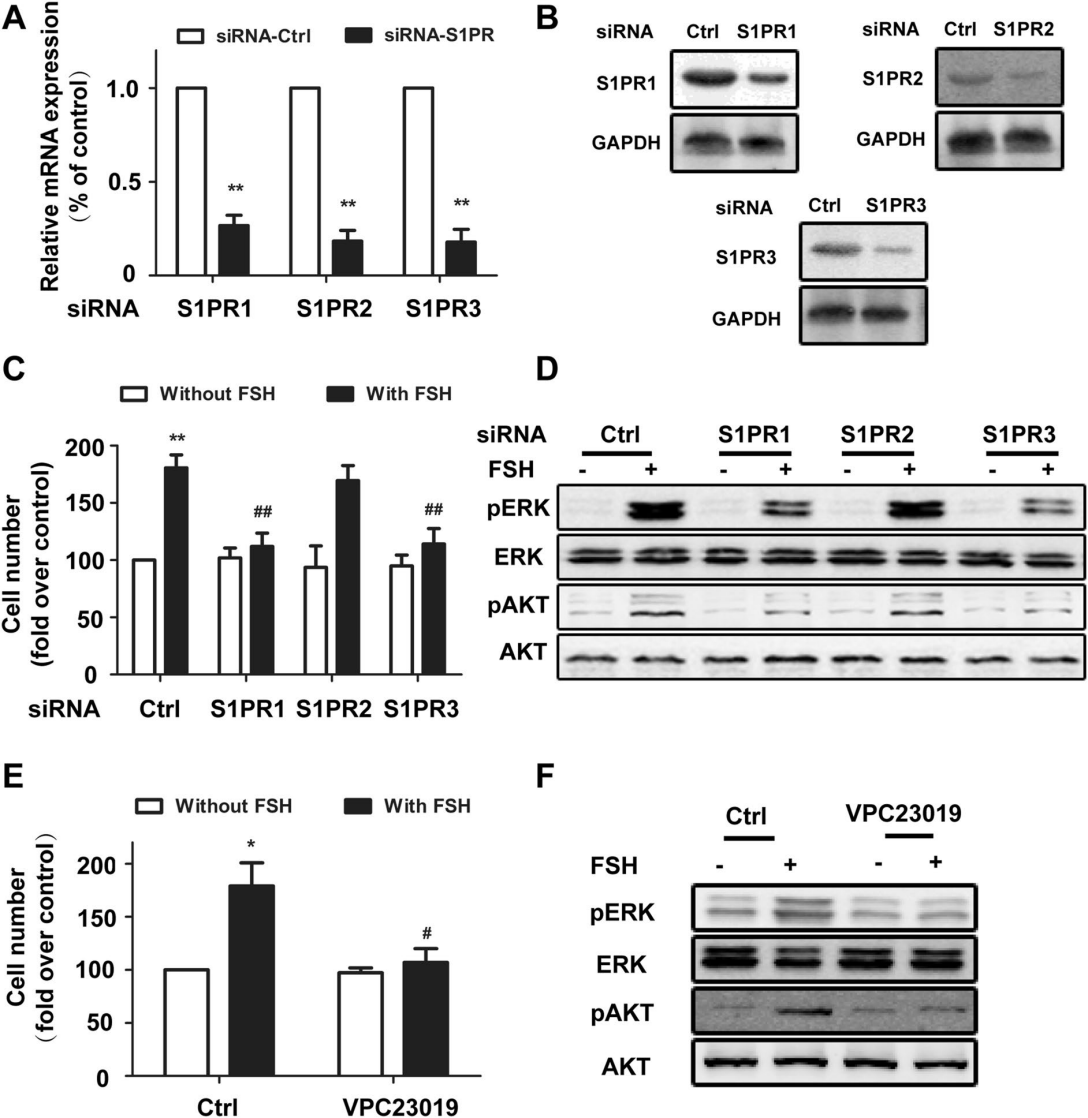
S1PR1 and S1PR3 are responsible for the proliferation stimulated by FSH in EOC. (**A**) HO8910 cells were transfected with control siRNA and specific siRNA targeting S1PR1-3 as indicated. After 24 h of transfection, mRNA levels of S1PR1-3 were determined by quantitative RT-PCR. Data are expressed as the change with respect to control siRNA. (**B**) After 48 h of transfection, protein levels of S1PR1-3 were determined by Immunoblotting analysis and normalized to GAPDH. (**C**) Having been transfected with the different siRNA, HO8910 cells were cultured with or without FSH (40 mIU/ml). After 72 h, cell survival ability was detected. (**D**) Cells transfected with specific siRNA were stimulated with or without FSH (40mIU/ml) for 30 min. Levels of pAkt and pErk were determined by Immunoblotting analysis. (**E**) Serum-starved HO8910 cells were pretreated by VPC23019 (300 nM) for 2 h, HO8910 cells were cultured with or without FSH (40 mIU/ml). After 72 h, cell survival ability was detected. (**F**) HO8910 cells having been pretreated with VPC23019, were treated with FSH (40 mIU/ml) for 30 min. Phosphorylated-Erk (pErk) and phosphorylated Akt (pAkt) were observed by immunoblotting analysis. Data are mean ± SD. *P < 0.05 vs. control/-FSH; #*P* < 0.05 vs. control/ + FSH.

SphK activation could catalyse the generation of sphingosine-1-phosphate (S1P), and S1P functions through S1P receptors to exert its biological effects. Because S1PR1-3 are widely expressed in every cell, we focused on investigating the role of S1PR1-3 in FSH-mediated proliferation through a SphK-mediated pathway. We applied specific siRNAs to knock down S1PR1, S1PR2, and S1PR3 expression. Notably, downregulating S1PR1 and S1PR3 expression (Fig. 6A,B) significantly inhibited FSH-induced proliferation of HO8910 cells (Fig. 6C). By contrast, cell proliferation was not significantly influenced by siRNA-mediated knockdown of S1PR2. In addition, siRNA-mediated knockdown of S1PR1 and S1PR3 resulted in a significant reduction in FSH-induced Erk and Akt phosphorylation (Fig. 6D). For further improve the results, we also used S1PR1/3 inhibitor VPC23019 to inhibit S1PR1 and SP1R3 in EOC cells. We observed that inhibition of S1PR1/3 with VPC23019 resulted a significant reduction in FSH-induced Erk and Akt phosphorylation (Fig. 6E,F). Similar results were also observed in HEY cell line (data not shown). Therefore, these data suggested that S1PR1 and S1PR3 are involved in the proliferation stimulated by FSH in HO8910 cells.

### FSHR is required for FSH-mediated SphK activation and Erk/Akt activation in EOC

**Figure 7:**
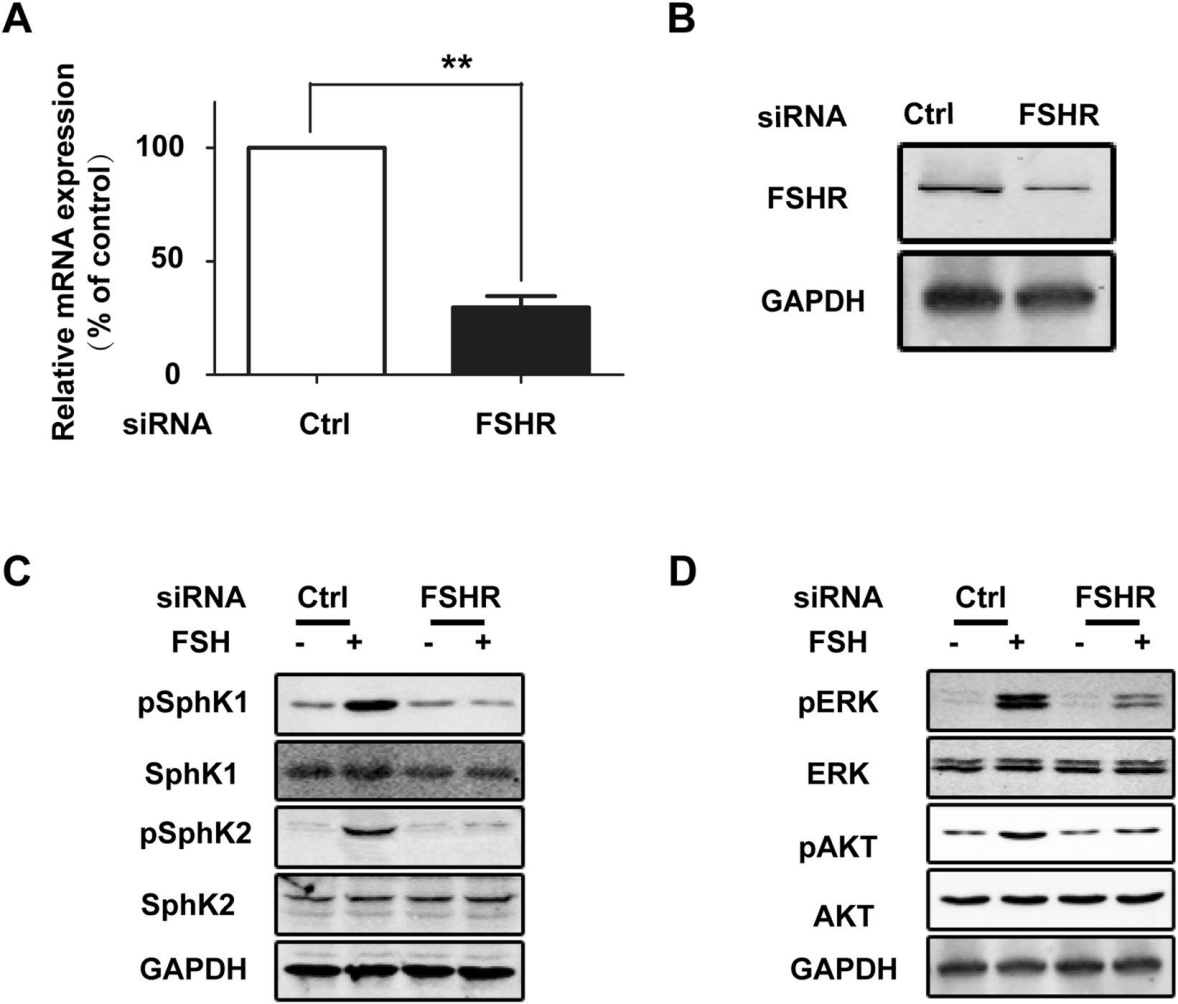
FSHR is required for FSH-mediated SphK activation and Erk/Akt activation in EOC. (**A**) HO8910 cells were transfected with control siRNA and specific siRNA targeting FSHR as indicated. After 24 h of transfection, mRNA levels of FSHR were determined by quantitative RT-PCR. Data are expressed as the change with respect to control siRNA. (**B**) After 48 h of transfection, protein levels of FSHR were determined by Immunoblotting analysis and normalized to GAPDH. (**C**) Having been transfected with the different siRNA, HO8910 cells were cultured with or without FSH (40 mIU/ml). Levels of pSphK1 and pSphK2 were determined by Immunoblotting analysis. Densitometric analysis of pSphK1 and pSphK2 corresponding to the bands were shown in the histogram (normalized to total SphK1 and SphK2). (**D**) Cells transfected with specific siRNA were stimulated with or without FSH (40mIU/ml) for 30 min. Levels of pAkt and pErk were determined by Immunoblotting analysis. Data are mean ± SD. *P < 0.05 vs. control/-FSH; #*P* < 0.05 vs. control/ + FSH.

Studies on the mechanism of action of FSH indicated that the gonadotropin receptor corresponding to FSH is a G protein-coupled receptor. Many studies have confirmed that abnormal function of the FSHR could lead to the development of ovarian cancer^3,25,27–29^. We sought to determine whether FSHR was involved in FSH-induced SphK activation. In our study, as shown in Fig. 7A,B, FSHR expression was significantly reduced in HO8910 cells with siRNA targeting FSHR. Compared with control cells, cells treated with FSHR-targeted siRNA showed a significant attenuation of FSH-induced phosphorylation of both SphK1 and SphK2 (Fig. 7C). Furthermore, phosphorylation of Erk and Akt induced by FSH was significantly inhibited by FSHR-siRNA (Fig. 7D). Similar results were also observed in HEY cell line (data not shown). These results, together with our observation that SphK1 and/or SphK2 blockage resulted in an inhibition of Erk and Akt activation, suggested that FSHR might modulate Erk and Akt signaling through SphK1/2.

## Discussion

In the current study, we provided the mechanistic data delineating a new pathway which mediated the mitogenic actions of FSH on EOC cells via SphK activation. SphK inhibition significantly suppressed FSH-dependent cell proliferation and clonogenic growth of EOC cells. Moreover, we found that SphK1 and SphK2, two isoenzymes always with opposing effects on cell survival, were both responsible for mediating FSH-induced EOC cell growth. This would suggest a new strategy that targets both isoenzymes of SphK for the management of EOC.

The two isoforms of SphK, SphK1 and SphK2, are involved in regulating physiological and pathological processes, including cancer progression^30,31^. Enhanced intra-cellular signaling through SphK1 was proposed as a mechanism responsible for hyper-proliferation and cell survival^32–34^. It has been widely suggested that the oncogenic properties of SphK1 are associated with “gain of function”. “Gain of function” may occur through SphK1-induced S1P stimulation mediated by agonists such as, growth factors, estradiol and insulin^21,35^. It is reported that intra-cellular dysregulation of SphK1 phosphorylation and localization were key elements in the transformation of the malignant phenotype^36,37^. In contrast to the well-studied oncogenic roles of SphK1 in cancer progression, the roles of SphK2 remain controversial for both pro-apoptotic and pro-survival functions have been suggested^38–40^. In this study, through Kaplan–Meier survival analysis, we revealed that for patients with EOC, both higher phospho-SphK1 and phospho-SphK2 expression were statistically associated with poorer prognosis. These results suggested that both SphK1 and SphK2 might play tumor promotion roles in EOC. Importantly, we also found that the levels of phospho-SphK1 and phospho-SphK2 protein were positively correlated with FSHR expression in EOC tissues, which suggested that there might be a certain regulatory relationship between FSH and SphK1/2 in EOC. Indeed, FSH stimulation induced a transient and rapid increase in phosphorylation of SphK1/2 in a dose dependent manner. Moreover, FSHR down-regulation profoundly blocked FSH-mediated phosphorylation of SphK1/2. These results suggested that FSH could activate SphK1/2 in EOC cells via FSHR. It is reported that FSH could promote the growth of EOC cells^23,25,41^. Besides, previous reports and our previous studies showed that both SphK1 and SphK2 are responsible for EOC proliferation^13,14,17,42^. However, the roles of SphK1/2 in FSH-induced proliferation of EOC remained unknown. In this study, we showed drug inhibition of SphK1/2 significantly suppressed FSH-dependent EOC cell growth. Moreover, FSH-mediated EOC proliferation could significantly inhibited by either SphK1 or SphK2 down-regulation. Together, these results suggested SphK1/2 was not only activated by FSH but also responsible for FSH-induced proliferation in EOC. This is consistent with our previous report showing that both SphK1 and SphK2 are important for insulin-induced growth in breast cancer cells^21^. These findings, together with other reports showing the pro-apoptotic effects of SphK2^43,44^, demonstrated that SphK2 regulated cell survival in a highly cell type-dependent manner.

The potential mechanism leading to SphK activation came from the studies demonstrating that Erk1/2-mediated phosphorylation is essential for both SphK1^45,46^ and SphK2 activation^47^. Given that Erk1/2 signaling is involved in FSH-induced multiple biological functions, we investigated its role in FSH-induced phosphor-SphK expression. Indeed, treatment with U0126, a specific MAPK/ERK kinase inhibitor, attenuated FSH-induced phosphorylation of both SphK1 and SphK2, indicating an important role of Erk in activating SphK1/2. It is well established that the activation of Erk and Akt pathways always plays a central role in the mitogenic effects of FSH^24,25^. Moreover, Akt and Erk are also downstream targets of SphK1/2 signaling^48^. Thus, our observation that FSH could activate SphK1/2, together with our finding that SphK1/2 is involved in FSH-induced cell proliferation, raised the possibility that the SphK signaling pathway may be involved in FSH activation of Erk and/or Akt. This idea is supported by the result that FSH-induced activation of Erk and Akt are partly abrogated by either SphK1 or SphK2 knockdown. These results, together with our observation that Erk blockage resulted in a significant inhibition of SphK1/2 activation induced by FSH, suggest that Erk could be placed both upstream and downstream of the SphK1/2 signaling and has a dual role in the initiation and amplification of the SphK1/2 signaling loop in ovarian cancer cells.

The biological consequences of SphK activation, mainly through phosphor-SphK1 and phosphor-SphK2^18,19^, chiefly rely on the actions of its product, S1P. In our previous studies, we confirmed that increased phospho-Sphk1 or phospho-SphK2 was correlated with the increased S1P in breast cancer cells^21^. Recently, we also confirmed increased phosphe-SphK1 correlating with increased S1P in ovarian cancer cells^13^. S1P functions mainly through activating a group of G-protein-coupled receptors, including S1PR1 to S1PR5, in an autocrine or paracrine manner^49^. Consistent with this “inside-out” signaling, our results showed that down-regulating S1PR1 or S1PR3, the predominantly expressed S1P receptors in EOC cells^13^, could partly block FSH-induced cell proliferation, Erk and Akt activation. This is in agreement with previous studies showing that S1PR1/3 is involved in Erk and Akt activation^50^ and cell proliferation^51^. However, knockdown of S1PR2, another over-expressed S1P receptor in EOC^13^, had almost no effect on FSH-stimulated EOC. Therefore, S1PR1 and S1PR3, but not S1PR2, are involved in FSH-induced proliferation in EOC. Nevertheless, until now the intracellular actions and targets of S1P have not been fully defined and further investigation is necessary. Furthermore, despite S1PR1 to S1PR3 being previously reported as^13^, and shown to be, the predominant receptors that accounts for the receptor-dependent action of S1P in EOC cells, we are unable to rule out the possibility that other members of the S1P receptor family expressed in the cell line may also be involved in the FSH-induced Erk and Akt activation and cell proliferation in EOC cells. This requires further investigation.

## Conclusion

In conclusion, for the first time, it has been demonstrated that SphK1 and SphK2 are involved in the FSH-mediated proliferation of EOC cells and play equivalent roles. Moreover, FSH-induced proliferation via SphK might function in the activation of the Erk and Akt pathways. Therefore, these findings suggest a novel mechanism for the proliferation of EOC cells stimulated by FSH. It might eventually serve as a new prognostic marker and permit the development of a new strategy that specifically targets both SphK1 and SphK2 for the prevention and treatment of EOC.

## Supplementary information

Supplementary Information.

## Abbreviations

FSH: Follicle-stimulating hormone
EOC: Epithelial ovarian cancer
FIGO: International Federation of Gynecology and Obstetrics
SphK: Sphingosine kinase
FSHR: Follicle-stimulating hormone receptor
S1P: Sphingosine-1-phosphate
S1PR: S1P receptor
ATCC: American type culture collection
SI: Staining index
OS: Overall survival
