# Supplementary material for: Follicle-stimulating hormone promotes the proliferation of epithelial ovarian cancer cells by activating sphingosine kinase
Source: Sci Rep. 2020 Aug 14;10:13834. doi: 10.1038/s41598-020-70896-0 (PMC7428003; doi:10.1038/s41598-020-70896-0)
Supplement: Supplementary file 1 — Supplementary Information. [file 41598_2020_70896_MOESM1_ESM.pdf]

# Follicle-stimulating hormone promotes the proliferation of epithelial ovarian cancer cells by activating sphingosine kinase

Keqi Song[1, 2¶], Lan Dai[1, 2¶*], Xiaoran Long[1, 2¶], Wenjing Wang[1, 2], Wen Di[1, 2, 3*]

[1]Department of Obstetrics and Gynecology, Ren Ji Hospital, School of Medicine, Shanghai Jiao Tong University, Shanghai 200127, China

[2]Shanghai Key Laboratory of Gynecologic Oncology, Shanghai 200127, China

[3]State Key Laboratory of Oncogene and Related Genes, Shanghai Cancer Institute, Ren Ji Hospital, School of Medicine, Shanghai Jiao Tong University, Shanghai 200127, China

[*]Corresponding authors:

E-mail: delta496@126.com (LD)

E-mail: diwen163@163.com (WD)

[¶]These authors contributed equally to this work.

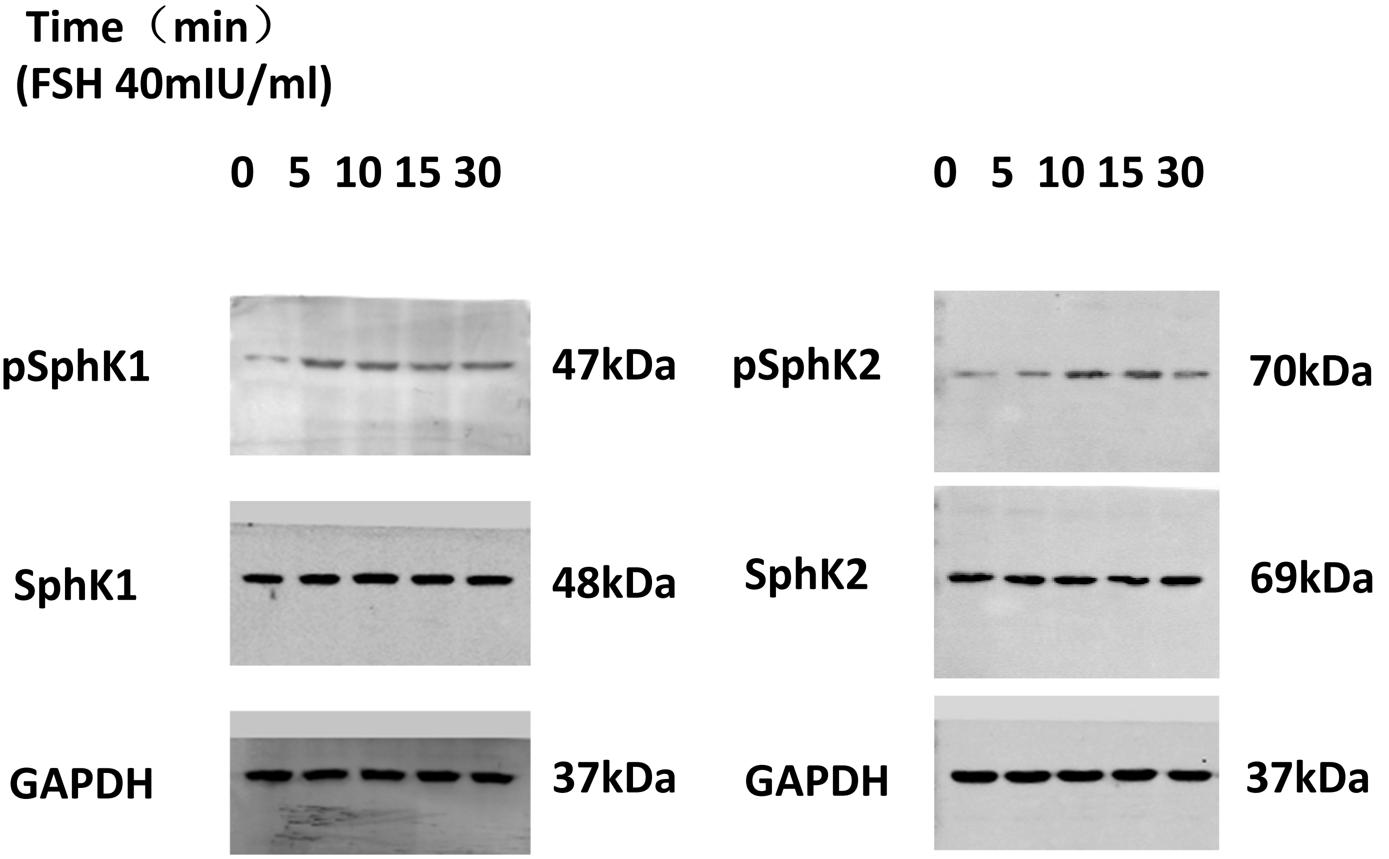


Fig S1 Full length western blot of Figure 3A

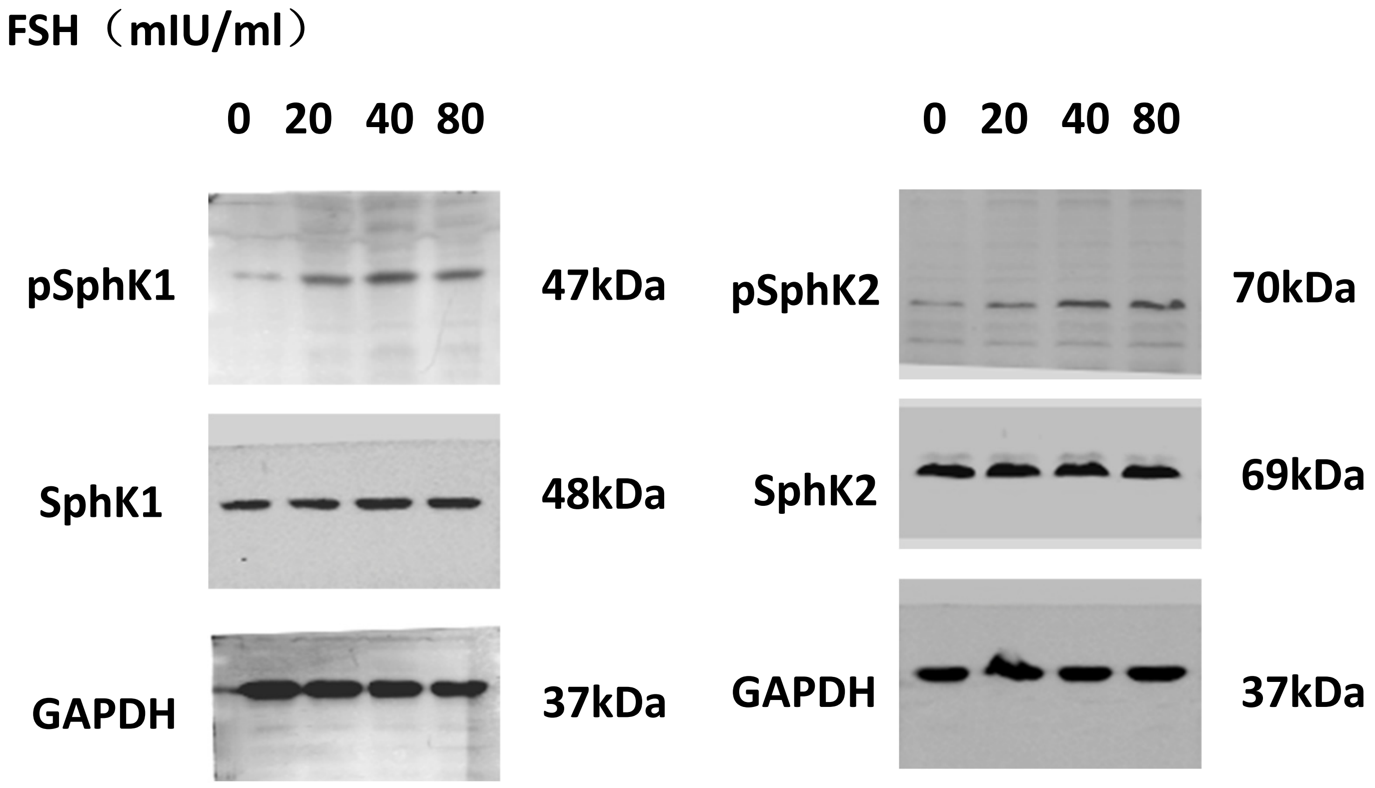


Fig S2 Full length western blot of Figure 3B

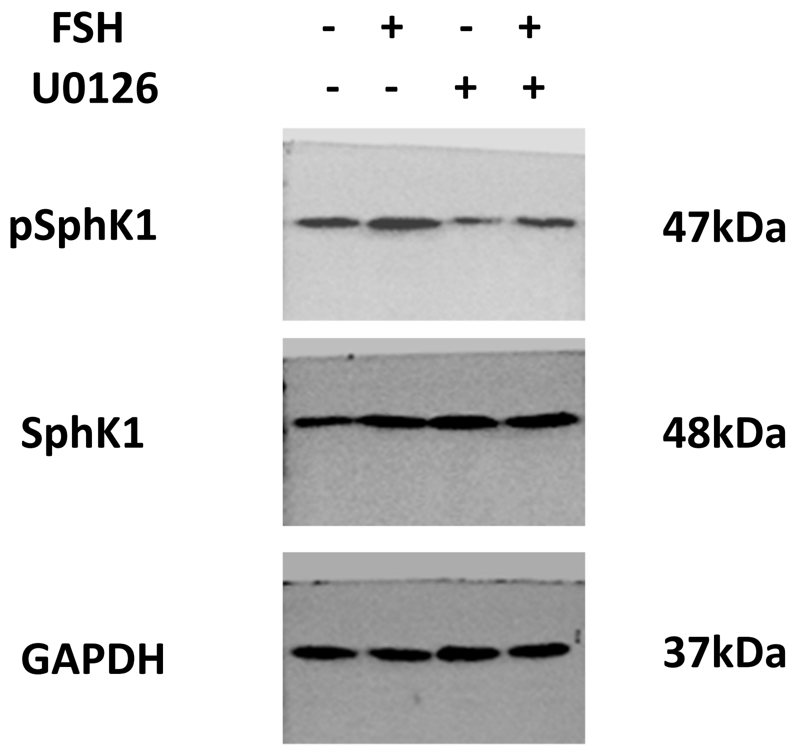


**Fig S3 Full length western blot of Figure 4A**

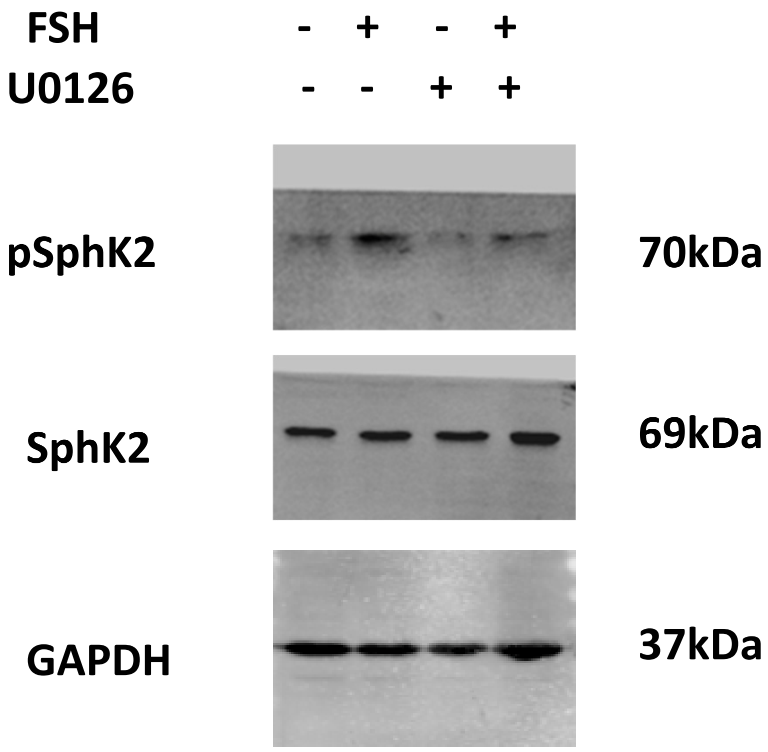


Fig S4 Full length western blot of Figure 4B

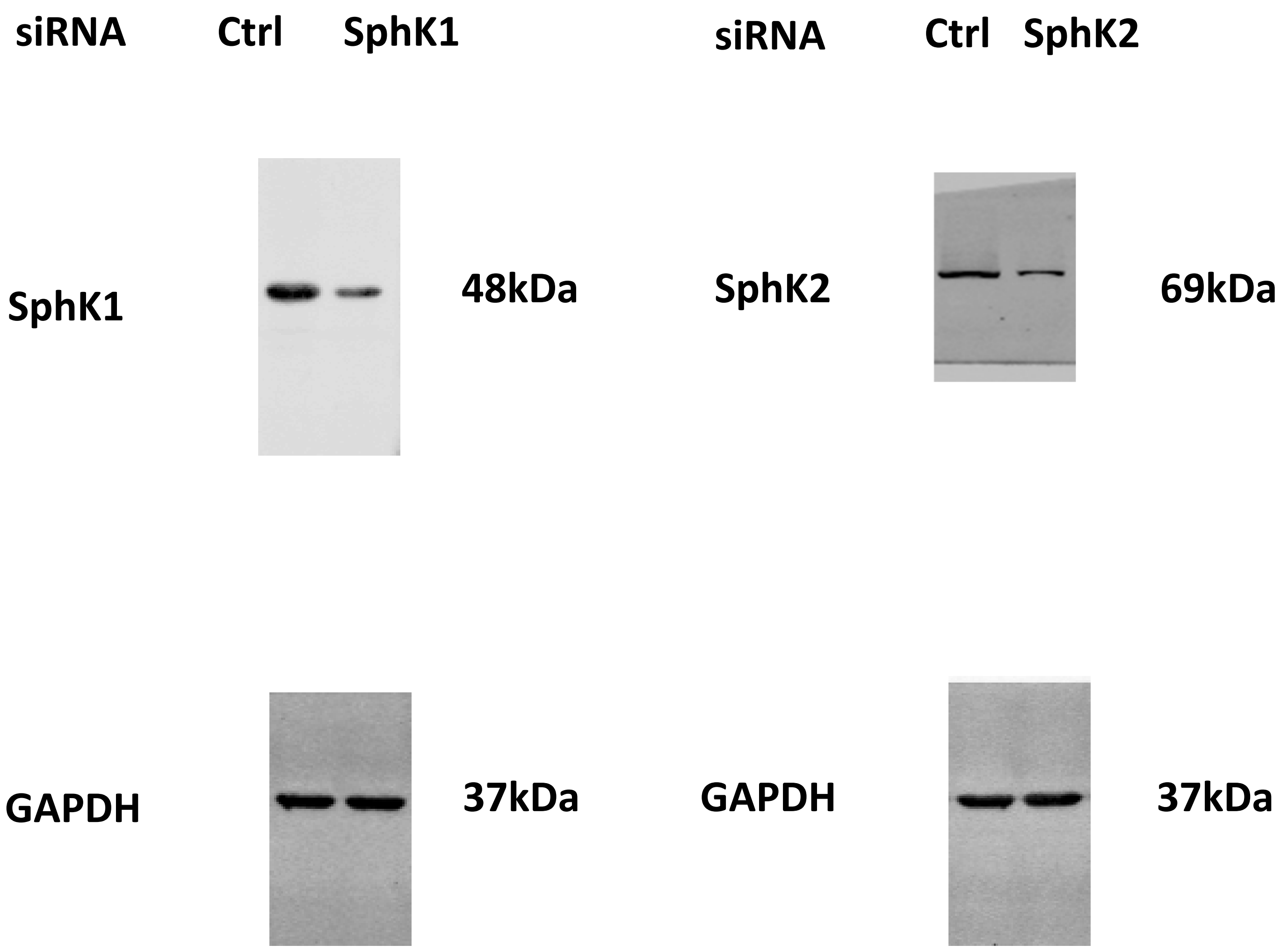


**Fig S5 Full length western blot of Figure 5B**

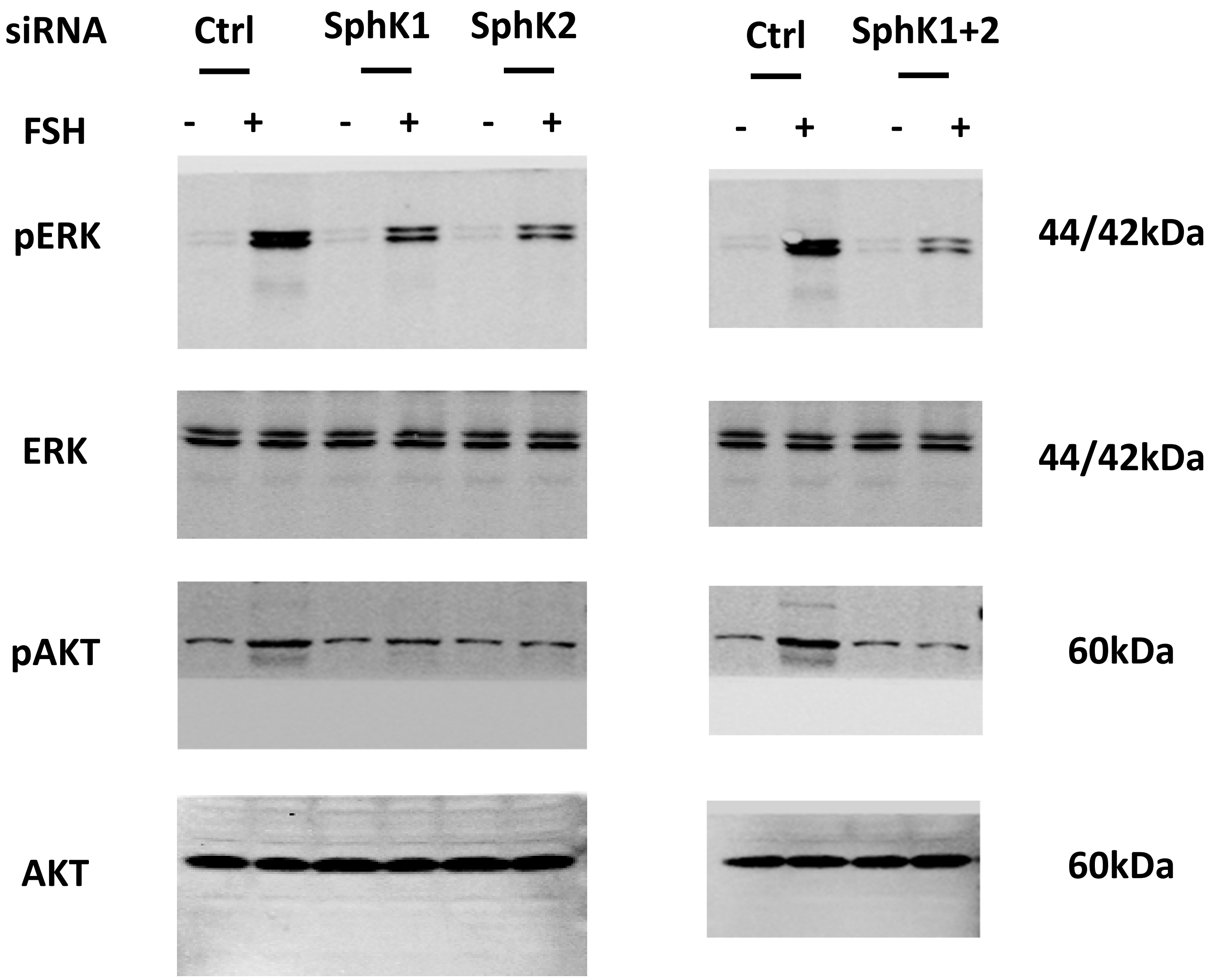


Fig S6 Full length western blot of Figure 5D

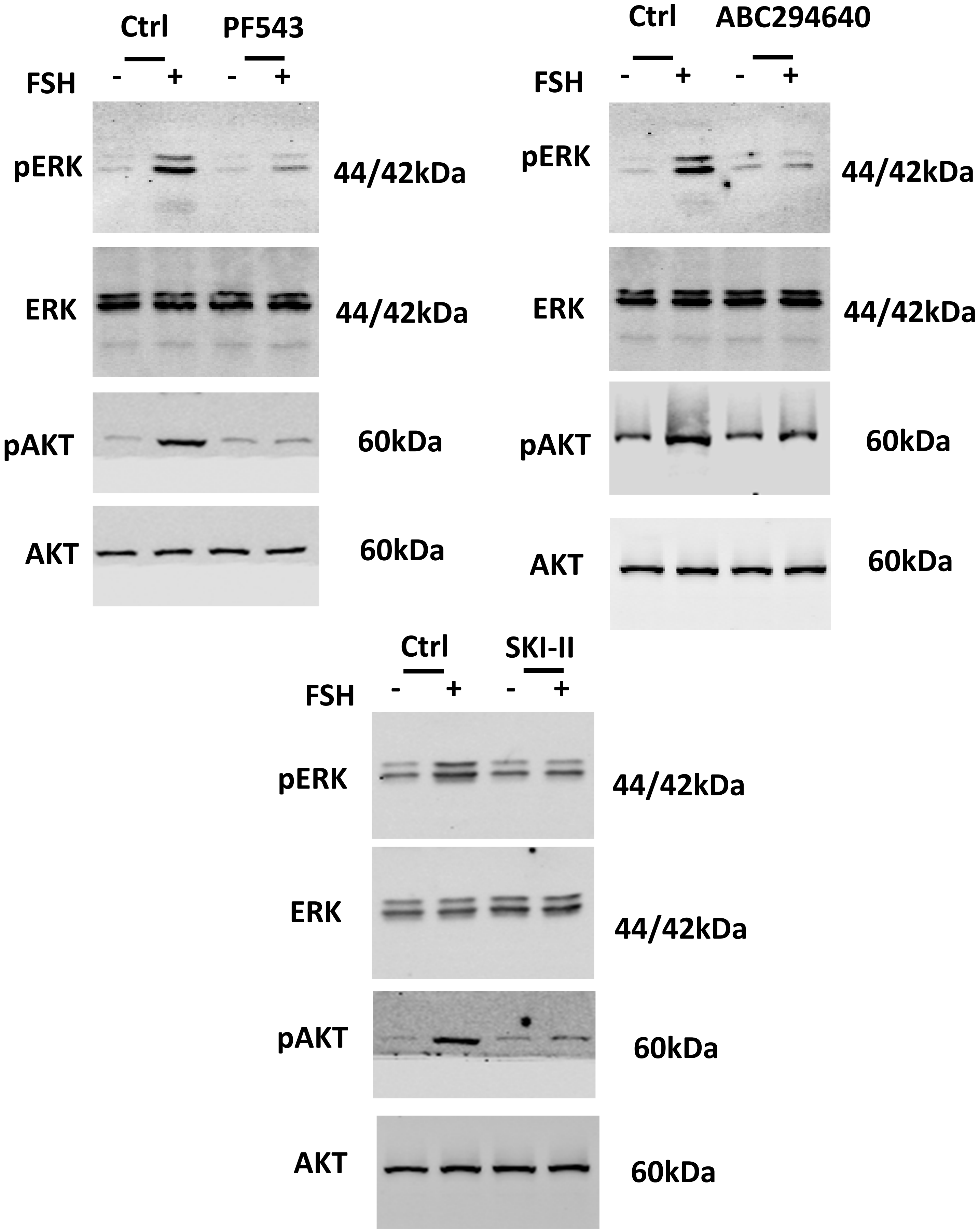


Fig S7 Full length western blot of Figure 5F

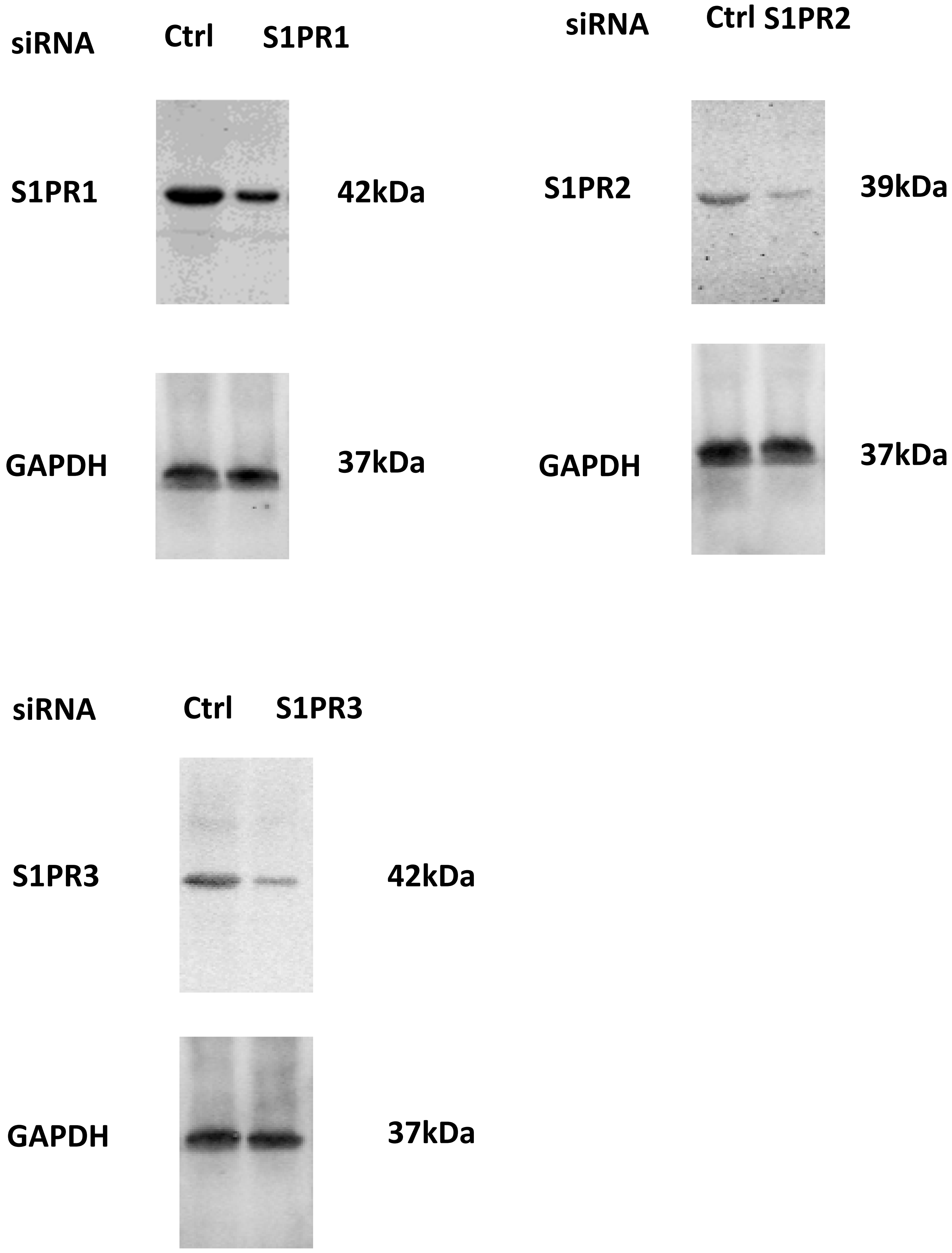


**Fig S8 Full length western blot of Figure 6B**

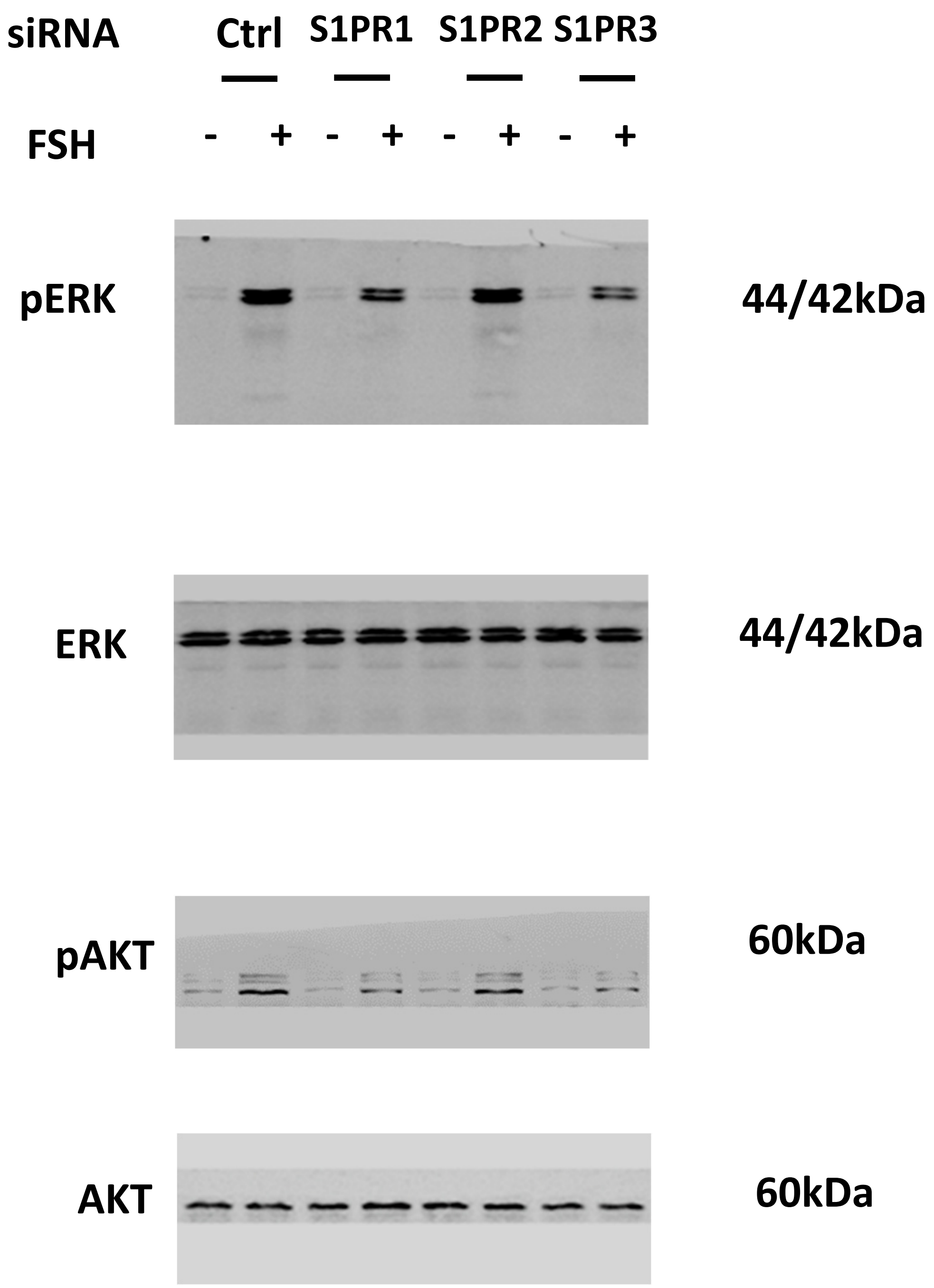


Fig S9 Full length western blot of Figure 6D

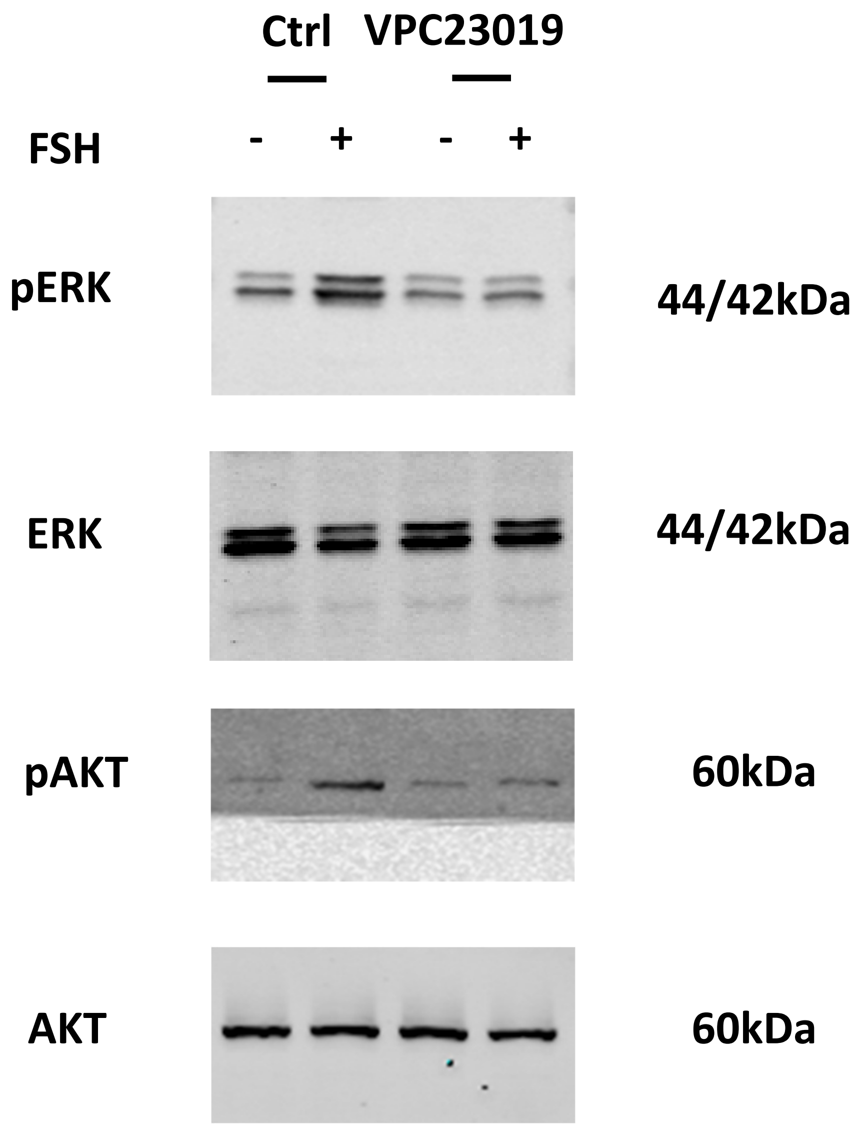


Fig S10 Full length western blot of Figure 6F

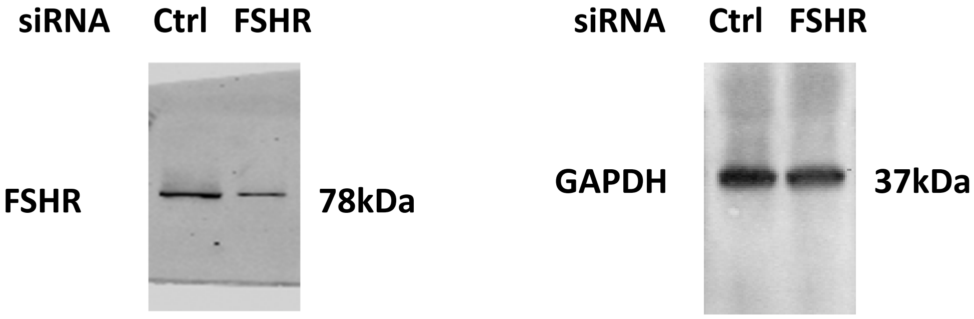


Fig S11 Full length western blot of Figure 7B

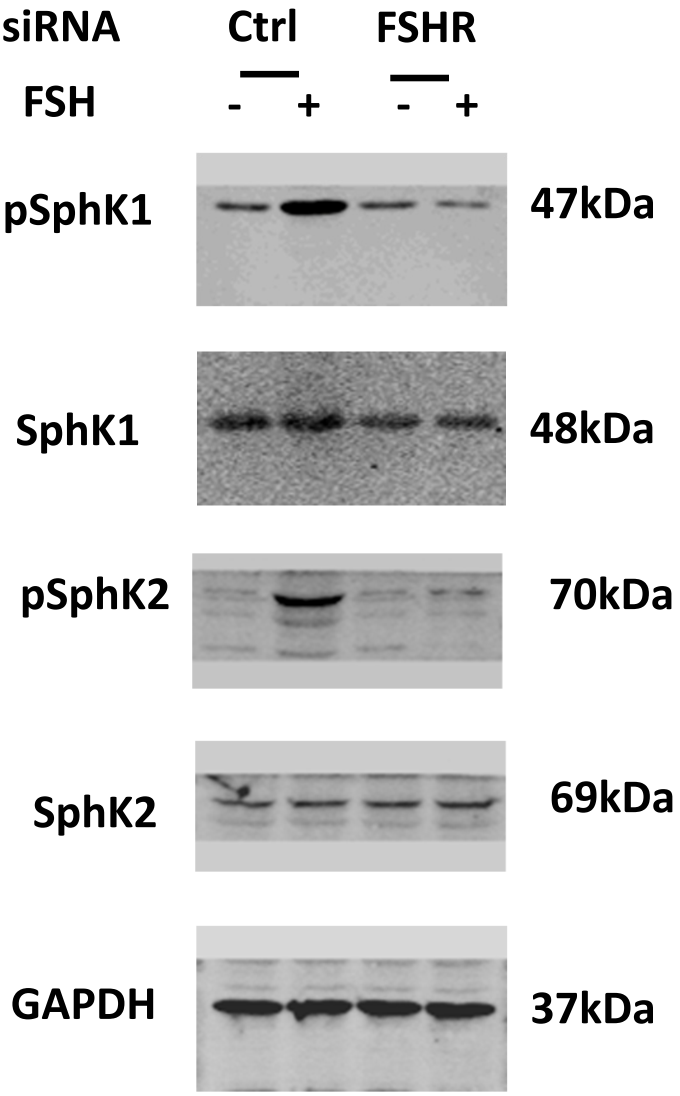


Fig S12 Full length western blot of Figure 7C

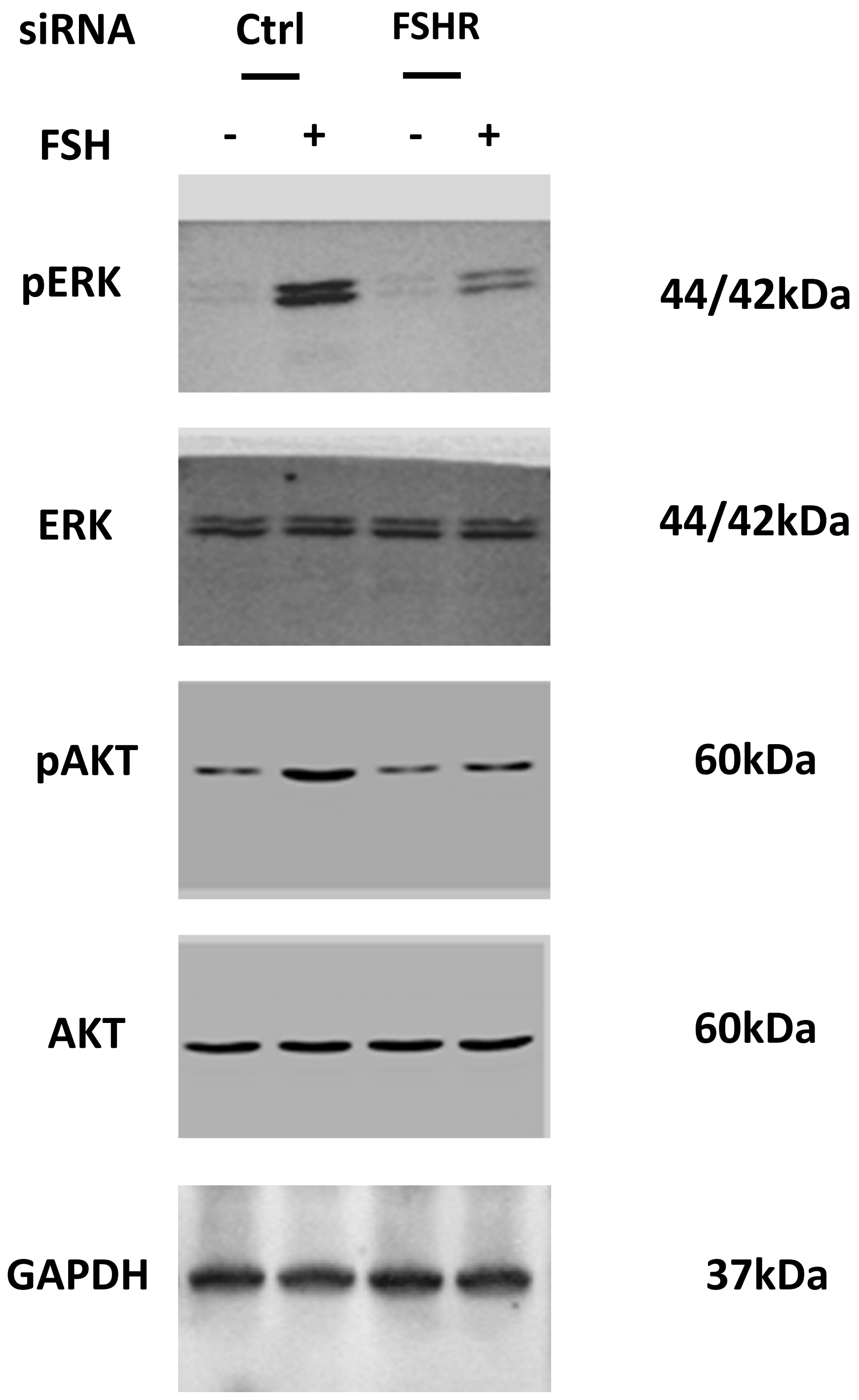


Fig S13 Full length western blot of Figure 7D
